# OPDAylation of Thiols of the Redox Regulatory Network In Vitro

**DOI:** 10.3390/antiox11050855

**Published:** 2022-04-27

**Authors:** Madita Knieper, Lara Vogelsang, Tim Guntelmann, Jens Sproß, Harald Gröger, Andrea Viehhauser, Karl-Josef Dietz

**Affiliations:** 1 Biochemistry and Physiology of Plants, Faculty of Biology, Bielefeld University, 33615 Bielefeld, Germany; madita-knieper@web.de (M.K.); lara.vogelsang@uni-bielefeld.de (L.V.); andrea.viehhauser@uni-bielefeld.de (A.V.); 2 Industrial Organic Chemistry and Biotechnology, Faculty of Chemistry, Bielefeld University, 33615 Bielefeld, Germany; tim.guntelmann@uni-bielefeld.de (T.G.); j.spross@uni-bielefeld.de (J.S.); harald.groeger@uni-bielefeld.de (H.G.)

**Keywords:** *Arabidopsis thaliana*, oxylipin, posttranslational modification, thiol, thioredoxin

## Abstract

cis-(+)-12-Oxophytodienoic acid (OPDA) is a reactive oxylipin produced by catalytic oxygenation of polyunsaturated α-linolenic acid (18:3 (ω − 3)) in the chloroplast. Apart from its function as precursor for jasmonic acid synthesis, OPDA serves as a signaling molecule and regulator on its own, namely by tuning enzyme activities and altering expression of OPDA-responsive genes. A possible reaction mechanism is the covalent binding of OPDA to thiols via the addition to the C=C double bond of its α,β-unsaturated carbonyl group in the cyclopentenone ring. The reactivity allows for covalent modification of accessible cysteinyl thiols in proteins. This work investigated the reaction of OPDA with selected chloroplast and cytosolic thioredoxins (TRX) and glutaredoxins (GRX) of *Arabidopsis thaliana*. OPDA reacted with TRX and GRX as detected by decreased m-PEG maleimide binding, consumption of OPDA, reduced ability for insulin reduction and inability to activate glyceraldehyde-3-phosphate dehydrogenase and regenerate glutathione peroxidase (GPXL8), and with lower efficiency, peroxiredoxin IIB (PRXIIB). OPDAylation of certain protein thiols occurs quickly and efficiently in vitro and is a potent post-translational modification in a stressful environment.

## 1. Introduction

Three chloroplast enzymes convert α-linolenic acid (18:3 (ω−3)) to *cis*-(+)-12-oxophytodienoic acid (OPDA) [1,2]. OPDA is exported from the chloroplast to the cytosol possibly employing the inner envelope protein OPDAT1 [3] and the outer envelope channel JASSY [4]. Following import into the peroxisome, OPDA reductase 3 (OPR3) and three cycles of ß-oxidation generate jasmonic acid (JA) [1,2]. An alternative route uses cytosolic OPR2 for the reduction of 4,5-didehydro-JA [5].

Apart from being a precursor for the plant hormone jasmonic acid, OPDA functions as a signaling compound and regulator of plant processes by two mechanisms, as reversibly binding ligand and as reagent covalently modifying target proteins at exposed cysteinyl thiols. An example for the OPDA function as a ligand is the chloroplast cyclophilin 20-3 (CYP20-3), which upon reversible binding of OPDA associates with the cysteine synthase complex and activates synthesis of O-acetyl-serine from serine, the precursor for cysteine synthesis, by serine acetyltransferase [6]. Reversibility of OPDA association with CYP20-3 independent of thiols was demonstrated by titrating free thiols after incubating CYP20-3 with thiol reactive compounds such as methyl vinylketone and OPDA [7]. While thiols of CYP20-3 were blocked with vinylketone, they remained reactive in the presence of OPDA.

On the other hand, cyclopentenone moieties-containing compounds react with thiols of glutathione and cysteine, but also cysteinyl thiols exposed on protein surfaces [7,8,9], similar to prostaglandins of the cyclopentenone type, such as 15dPGJ2 and PG-A1. Dückershoff et al. [8] infiltrated *Arabidopsis thaliana* leaves with 100 µM OPDA or phytoprostanes for 6 h and identified 66 polypeptides with altered abundance in 2D gels by comparative image analysis and mass spectrometric identification. Among these proteins were 13-LOX 2, CYP 20-3, thioredoxin m4 and peroxiredoxin Q. Infiltration of biotinylated PG-A1 followed by affinity purification failed to unequivocally identify proteins, but nevertheless confirmed the binding of PG-A1 to many polypeptides. Furthermore, the authors showed OPDAylation of recombinant glutathione-S-transferase GST1 and GST6 [8]. In a subsequent analysis, OPDAylation was also shown for the transcription factor TGA2 at Cys186. However, this Cys was not needed for transcriptional activation of TGA2-dependent genes [9]. This pioneering proof of concept of OPDAylation defined starting points and identified present day limitations. Exogenous application of oxylipins likely establishes different subcellular oxylipin concentrations and, therefore, different polypeptides may be OPDAylated qualitatively and quantitatively in this approach with exogenous feeding compared to the in vivo situation. The 2D gel-based identification of target polypeptides combined with the, at that time, available mass spectrometry only focused on abundant proteins.

Shibata et al. [10] demonstrated the electrophilic character and reactivity of 15d-PGJ2 and its ability for covalent binding to human TRX-1. Incubation of SH-SY5Y cells with 25 µM or 50 µM 15d-PGJ2 induced oxidative stress as visualized by 2′,7′-dichlorodihydrofluorescein fluorescence. Overexpressing TRX1 decreased the oxidative stress in the cells treated with 15d-PGJ2 [10].

Covalent modification of protein thiols by reaction with OPDA was termed OPDAylation [7] in analogy to the nomenclature of other post-translational modifications such as glutathionylation or sumoylation. Maynard et al. reported preliminary evidence that chloroplast TRX-f1 incubated with OPDA is OPDAylated in vitro, and that OPDAylated TRX-f1 loses its function to reductively activate chloroplast fructose-1,6-bisphosphatase, one of its preferred targets [7]. The possibility of OPDAylation of proteins of the redox regulatory network in plants has not been addressed in any detail. For this reason, this study on protein thiol reactivity with OPDA was designed with focus on central elements of the redox regulatory network of the cytosol and chloroplast.

Protein thiols play decisive roles in adjusting protein activities. Diverse post-translational modifications occur on cysteinyl thiols, e.g., disulfide formation, sulfenylation, sulfinylation, sulfonylation, S-nitrosylation, S-glutathionylation and persulfidation [11]. Cys modification alters protein function and this mechanism was denominated Cys or thiol switch. In plants thiol switching plays a profound role in regulating various processes in different subcellular compartments and this research area is an expanding field [12]. The regulatory thiol-redox network consists of functional proteins that can be grouped into the categories of redox input elements, redox transmitters, redox targets and redox sensors [13]. Redox transmitters comprise TRX and glutaredoxins (GRX) which have evolved to large gene families in plants with multiple functions [14,15]. TRXs function in both directions of target protein regulation, namely as disulfide reductases and dithiol oxidases [16]. In the light of the central function of TRXs and GRXs in process regulation, it is important to understand whether and by which mechanisms their activity might be controlled apart from gene expression.

This work builds on the described evidence from work with 15d-PGJ2 and its ability to bind to human TRX1 [10] and the preliminary data on chloroplast TRX-f1 [7] and aimed to explore the propensity of other TRXs to react with OPDA and to become OPDAylated. Selected *A. thaliana* plastidic and cytosolic TRXs and GRXs were treated with OPDA and scrutinized for OPDAylation. Results from mPEG-maleimide binding, reduction assays with insulin, OPDA consumption, an activation test for glyceraldehyde-3-phosphate dehydrogenase and the reductive regeneration of glutathione peroxidase such as protein (GPXL) and type II peroxiredoxin in H_2_O_2_ peroxidase tests showed the effect of OPDAylation on these activities.

## 2. Materials and Methods

### 2.1. Cloning, Expression and Purification of Recombinant Proteins

All analyses were performed with purified recombinant proteins cloned from *Arabidopsis thaliana.* The coding sequences of *A. thaliana* TRXh3 (At5g42980), TRXh5 (At1g45145), GRXC2 (AT5G40370), GRXC5 (AT4G28730, without the sequence encoding for the transit peptide), GPXL8 (At1g63460), NTRA (At2g17420, without the sequence encoding for the transit peptide), PRXIIB (At1g65980), GR (At3g24170) and PGK3 (AT1G79550) were amplified from leaf cDNA using specific forward and reverse primers with restriction sites, if required (Table S1). TRXh3, TRXh5, GRXC2, GPXL8, NTRA, PRXIIB, GR and PGK3 were cloned into pET28a, GRXC5 in pEXP5-NT/TOPO. Sequences were verified by DNA sequencing. The expression vectors for TRXf1 (At3g02730) and TRXm4 (At3g15360) were cloned as described in Dreyer at al. [17]. GAPC2 (At1g13440) plasmid was kindly provided by the working group of Renate Scheibe [18].

For recombinant protein expression, *E. coli* NiCo21 (DE3) cells were transformed with plasmid DNA. After the induction of protein expression with 400 μM isopropyl-β-D-thiogalactopyranoside in the exponential phase, expression was carried out for 4 h at 37 °C and 170 rpm. Bacteria were harvested by centrifugation (11,000× *g*, 10 min, 4 °C) and resuspended in lysis buffer (50 mM Tris-HCl pH 8, 300 mM NaCl, 5 mM imidazole, 10 mM β-mercaptoethanol, 1 mM PMSF).

Cells were lysed by adding 200 µg/mL lysozyme for 30 min at 30 °C, followed by sonication and the soluble and insoluble fractions were separated by centrifugation at 20,000× *g* and 4 °C for 60 min. The lysate was incubated with Roti^®^Garose-His/Ni NTA-beads (Carl Roth, Karlsruhe, Germany) previously equilibrated with lysis buffer, under slow shaking at 4 °C for 60 min. After washing with 200 mL washing buffer I (50 mM Tris-HCl pH 8, 300 mM NaCl, 10 mM imidazole) and 50 mL washing buffer II (50 mM Tris-HCl pH 8, 300 mM NaCl, 20 mM imidazole), the His-tagged protein was eluted using the elution buffer (50 mM Tris-HCl pH 8, 300 mM NaCl, 250 mM imidazole, 10 mM β-mercaptoethanol, 1 mM PMSF). Eluted protein-containing fractions were pooled, concentrated and dialyzed over night at 4 °C against 40 mM K-Pi, pH 7.2 (Dialysis tubing Membra-Cel™, cellulose, flat width 25 mm, MWCO 14000, Carl Roth, range of weight of concentrated proteins: 14.21 kDa (GrxC2) to 56.32 kDa (GR)), with repeated changes of dialysis buffer. Final purity was checked by separation in 12% (*w/v*) SDS-PAGE.

Prior to usage, the proteins were centrifuged for 5 min at 20,000× *g* to remove protein aggregates. Protein concentration was determined spectrophotometrically using the protein-specific molar extinction coefficient at 280 nm calculated with the Expasy ProtParam tool [19].

### 2.2. Synthesis of OPDA

Synthesis of OPDA followed the method described in the literature starting from α-linolenic acid and utilizing 13-lipoxygenase, allene oxidase and allene oxide cyclase in accordance with the in planta biosynthesis. A representative procedure as well as literature references are given in Löwe et al. [20]. In this literature process, which also served as the basis for this sample preparation, a soybean lipoxygenase (SIGMA Chemicals) and whole cell catalysts from Escherichia coli expressing allene oxide synthase and allene oxide cyclase, both from *A. thaliana*, were used to convert α-linolenic acid to 12-OPDA. The chemical identity of cis-(+)-12-OPDA was proven by means of 1H-NMR spectroscopy and the accordance of the detected signals with those reported in literature for cis-(+)-12-OPDA [20]. The chemical purity was 98%, and diastereochemic ratio turned out to be d.r. (cis/trans) = 98:2. Both chemical purity and diastereomeric ratio were also determined by 1H-NMR spectroscopic analysis of the sample.

### 2.3. OPDA-Treatment of Recombinant Proteins

In total, 100 µM TRX or GRX was incubated with 10 mM DTT for 1 h at RT. The incubate was desalted by passage through a PD-10 column (GE Healthcare Life Sciences, Little Chalfont, UK). Afterwards, 20 µM desalted recombinant protein was treated with specified OPDA concentrations for indicated time periods. As a control, proteins were incubated with the solvent (EtOH), respectively.

### 2.4. Labelling of Free Thiol Groups and Electrophoretic Mobility Shift Assay

Recombinant proteins (TRX-h3, -h5, -f1, -m4 and GRX-C2) were prepared and treated with OPDA as described before. After 1 h of incubation, mPEGmal-5000 was added to 2 µg of protein to a final concentration of 1 mM. Samples were incubated at RT and 300 rpm for 1 h and directly mixed with 5× loading dye (225 mM Tris pH 6.8, 5% (*w*/*v*) SDS, 0.05% (*w*/*v*) bromophenol blue, 50% (*v*/*v*) glycerol, 200 mM DTT) and heated at 95 °C for 5 min.

Analysis of samples was performed by SDS-PAGE (separation at 50 mA for 80 min) and subsequent Coomassie brilliant blue staining. Pageruler Unstained Protein Ladder (Thermo Fisher Scientific, Waltham, Massachusetts, USA) was used as molecular mass standard.

### 2.5. Insulin Precipitation Assay for Assessment of Disulfide Reduction Activity

To analyze TRX and GRX disulfide reduction activity, recombinant protein was prepared as described before. A final concentration of 5 µM protein was added to the insulin reaction mixture (100 mM KPi, pH 7.2, 1 mM EDTA, 160 µM insulin) in a total volume of 200 µL [21]. Insulin (from bovine pancreas) was purchased from Merck (Darmstadt, Germany). Directly before monitoring the increase in turbidity at 650 nm, DTT was added to a final concentration of 1 mM. Absorbance was measured using a Biotek KC4 microplate reader with a time interval of 19 s.

### 2.6. HPLC Analysis of OPDA

Analysis of OPDA using rp-HPLC was carried out using a Dionex HPLC system and a LiChrospherR 100 RP-18 column (LiChrom-CARTR 250-4, Merck) at 30 °C and a flow rate of 1.75 mL/min. At the start of each run, the column was equilibrated for 8 min with solution A (80% MeOH, 19.9% H_2_O, 0.1% acetic acid). In total, 25 µL sample volume was injected into solution A. After 9 min, percentage of solution B (99.9% MeOH, 0.1% acetic acid) was gradually increased to 25%. After 25% was reached after 14 min, concentration of solution B was increased to 100% within 2 min (at 16 min). The run was stopped at 18 min, and regeneration of the column was achieved with solution A for 8 min. The UV spectrum of each run was recorded at 224 nm (Chromeleon Version 6.6, Dionex).

To determine the binding of OPDA to TRX-h3, -h5, -f1 and -m4, 200 µM of OPDA was incubated with either 50 or 100 µM of recombinant protein in a total volume of 40 µL. Control samples were prepared by incubation of OPDA with an equal volume of 0.1 M KPi, pH 7.2, instead of protein solution. After a minimum incubation time of 2 h at RT, samples were analyzed by rp-HPLC and the amount of OPDA bound to TRX was calculated by subtracting the remaining OPDA amount from the initial 8 nmol OPDA.

### 2.7. NADPH-Dependent Peroxidase Activity Measurement

The rate of H_2_O_2_ reduction by thiol-peroxidases was quantified using the TRX system as reductant in the case of GPXL8 and the GRX system in the case of PRXIIB. The activity was measured by monitoring NADPH oxidation by spectrophotometry at 340 nm (Cary 3500 UV-Vis, Agilent, Santa Clara, CA, USA). The assay was performed in quartz cuvettes with 3 μM thiol peroxidase, 200 μM NADPH and 5 mM EDTA in 0.1 M Tris-HCl, pH 7.5, at RT. Varying amounts of TRX in combination with 1 µM NTRA were tested for optimizing the TRX system. For optimizing the GRX system, 2 mM GSH and 1 µM GR were supplemented with varying amounts of GRX. The reaction mixture (100 µL) was incubated at 25 °C for 5 min. OPDA was added and incubation time adjusted as indicated. The assay was equilibrated for 3 min, the baseline recorded, the reaction initiated by the addition of 300 µM H_2_O_2_ and the absorption change monitored at 340 nm. Enzyme activities were calculated using an ε-value for NADPH of 6220 M^−1.^cm^−1^.

### 2.8. Thioltransferase Activity (HED Assay)

The hydroxyethyldisulfide (HED, Sigma-Aldrich) reduction activity was measured at RT by following the NADPH oxidation in the presence of the GRX reducing system. The 100 μL reaction mixture contained 200 µM NADPH, 2 mM GSH, 0.7 mM HED, 5 mM EDTA and 1 µM GR in 100 mM Tris-HCl, pH 7.5. After 5 min of preincubation at 25 °C, a baseline was recorded before 0.1 µM GRX was added to start the reaction. The decrease in absorbance was measured spectrophotometrically at 340 nm (Cary 3500 UV-Vis, Agilent, Santa Clara, CA, USA). Enzyme activities were calculated using a molar ε-value for NADPH of 6220 M^−1.^cm^−1^.

### 2.9. Measurement of Glyceraldehyde-3-Phosphate Dehydrogenase Activity

Recombinant His_10_-tagged GAPC2 (30 µM) was incubated with 140 µM NAD^+^ and 5 mM DTT at RT for 30 min. In total, 1 µM reduced and desalted (Zeba Spin Desalting Columns, Thermo Fisher Scientific, Waltham, MA, USA) GAPC2 and 140 µM NAD^+^ were mixed with 25, 50, 100 or 500 µM OPDA or the respective concentrations of solvent (EtOH), 50 µM H_2_O_2_ or 10 mM DTT, followed by a 1 h incubation at RT.

The reaction mixture containing 4 mM ATP, 8 mM 3-phosphoglyceric acid disodium salt (3-PGA), 0.294 mM NADH, 90 nM phosphoglycerate kinase (PGK), 8 mM MgSO_4_ and 1 mM EDTA in 100 mM Tris-HCl, pH 7.8, was preincubated for 10 min at RT, so that the GAPC2-substrate 1,3-bisphosphoglycerate was formed, and the mixture was degassed afterwards. The reaction mix was measured at RT in a quartz cuvette at 340 nm for 1–2 min before 15 nM pretreated GAPC2 was added. The spectrophotometric measurement was continued for further 5 min (Cary 3500 UV-Vis, Agilent, Santa Clara, CA, USA). Enzyme activity was calculated using the molar extinction coefficient of NADH (ε = 6220 M^−1.^cm^−1^).

### 2.10. Mass-Spectrometric Analysis of OPDAylated TRX-h3

The OPDAylated TRX-h3 was subjected to MS analysis. Following incubation with DTT as described above (2.4.) desalting was achieved with PD10 columns equilibrated with 50 mM ammonium acetate. In total, 20 µM TRX-h3 protein was incubated with 150 µM OPDA overnight and analyzed with a Q-IMS-TOF mass spectrometer Synapt G2Si (Waters GmbH, Manchester, UK) operated in resolution mode and interfaced to a nano-ESI ion source. N_2_ generated with the nitrogen generator NGM 11 served as nebulizer and dry gas. Argon was used as collision gas for MS2 experiments using the collision induced dissociation (CID). Samples were introduced by static nano-ESI using in-house pulled glass emitters. External calibration of the mass axis used ESI-L Tuning Mix (Agilent Technologies, Santa Clara, CA, USA) as standard. Scan accumulation and data processing was performed with MassLynx 4.1 (Waters GmbH, Manchester, UK) on a PC Workstation. The spectra were generated by accumulating and averaging 50 single spectra. Determinations of exact masses of OPDA were performed using centroided data using the protonated signal of leucine-enkephalin as an internal mass standard. Determination of protein masses was performed with the tool of Winkler [22]. Prior to protein mass determination, MS data were baseline subtracted, smoothed and centroided.

### 2.11. Plant Growth

*A. thaliana* Col0 plants were grown in soil in a greenhouse with a day/night cycle of 8 h/16 h, realized by automated shading, under natural winter irradiation (January/February) with 100 µmol photons^.^m^−2^ s^−1^ supplementary light (Philips Son-T Agro, high-pressure sodium lamp 400 W) at 19 °C/17 °C and 45–55% relative humidity. Rosettes were harvested at an age of 36–40 d.

### 2.12. OPDA Treatment of Leaf Discs

Leaf discs of 5 mm diameter were cut out and placed immediately on 100 µM CaCl_2_ solution (for membrane stabilization) containing 25, 50, 100, 250 or 500 µM OPDA or the respective concentrations of solvent (EtOH) as controls. Leaf discs were randomly placed either upside down or upside up. Finally, 96-well plates with floating leaf discs were closed with their lid and exposed to light (100 μmol photons m^−2^ s^−1^) or dark at 22 °C. The experiment was conducted three times.

### 2.13. Determination of Quantum Yield of Photosystem II

The effective quantum yield of PSII (ΔF/Fm‘) was measured under the respective light condition using the pulse-amplitude-modulated photosynthesis yield analyzer (Mini-PAM, Walz, Germany) [23].

### 2.14. Quantification of Protein, and Free Thiols

To determine the contents of proteins and non-protein thiols 7–8 leaf discs (appr. 20 mg FW) were ground in liquid nitrogen and extracted with Tris buffer (10 mM, pH 7,2). The excitation wavelength was set to 504 nm, the emission wavelength to 529 nm. Protein concentration was determined with the Bio-Rad assay. The thiol quantification method was based on the thiol reactivity of Ellman’s reagent (DTNB) using a standard curve generated with GSH [17].

### 2.15. Statistical Tests

If not indicated otherwise, significance of difference was determined using ANOVA, followed by post hoc Tukey test and is marked with letters.

## 3. Results

This work aimed to explore the reactivity of OPDA with plant thiol proteins, in particular with selected TRXs and GRX-C2. Following the synthetic pathway for one pot synthesis of OPDA from α-linolenic acid [20,24] we synthesized OPDA for use in this study. All proteins were molecularly cloned from *A. thaliana*, recombinantly produced in *E. coli* and purified to high purity as judged from silver-stained electropherograms.

### 3.1. Reaction of TRX Thiols with OPDA

#### 3.1.1. OPDA Effect on mPEG-Maleimide-Reactive Thiols

The first set of experiments aimed at investigating the reaction of OPDA with protein thiols using four different TRXs, the chloroplast TRX-f1 and TRX-m4 and the cytosolic TRX-h3 and Trx-h5, as well as the cytosolic GRX-C2.

**Figure 1:**
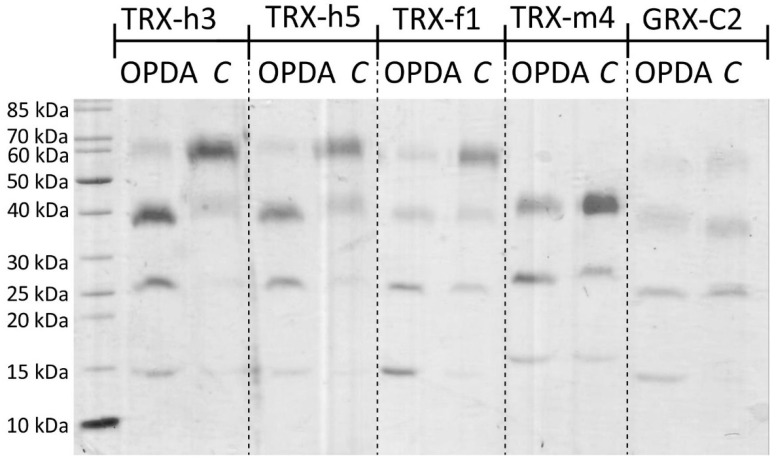
Blocking of exposed thiols of TRXs and GRXC2 by OPDA as indicated by less mPEGmal binding. Recombinant TRX-h3, TRX-h5, TRX-f1, TRX-m4 and GRX-C2 were reduced and desalted, and either treated with 150 µM OPDA or the equivalent amount of the solvent ethanol (“*C*”). Samples were treated with mPEG-maleimide 5000 to label free thiols. Shifts to increased apparent molecular mass indicates thiol accessibility for modification, while the lack of shifts indicates inaccessibility of thiols after treatment with OPDA. Similar results were seen in 4 experiments. Band quantification is presented in Supplementary Figure S1.

The electrophoretic mobility shift assay following the reaction of accessible thiols with mPEG-maleimide (mPEG_mal_) relies on an increase in molecular mass which slows down the migration in the SDS-PAGE. Figure 1 reveals the separation of TRX-h3 and -h5 at the expected molecular mass of 15 kDa. In the absence of OPDA, reaction with mPEG_mal_ shifted the prominent band to about 60 kDa. After preincubation with 150 µM OPDA prior to the mPEGmal treatment, the prominent band migrated at about 40 kDa. Lower binding stoichiometry of mPEGmal indicates that at least one cysteinyl thiol was blocked by OPDAylation. Additionally, the band at about 25 kDa increased in intensity. This result is interpreted that TRX-h3/5 had bound a second OPDA molecule. A similar pattern could be observed for TRX-f1 and GRX-C2 with the 25 kDa band being the most prominent one. Separation of TRX-m4 resulted in only three bands at roughly 17 kDa, 27 kDa and 40 kDa, irrespective of the treatment. Again, the band at roughly 27 kDa was the most intense one for protein incubated with 12-OPDA, while the intensity of the 40 kDa band intensified in the control sample. The results supported the conclusion that TRX thiols readily react with OPDA. Therefore, we addressed the disulfide exchange activity of TRXs and GRX-C2 with the established insulin reduction test.

#### 3.1.2. OPDA Effect on TRX-Dependent Insulin Reduction

**Figure 2:**
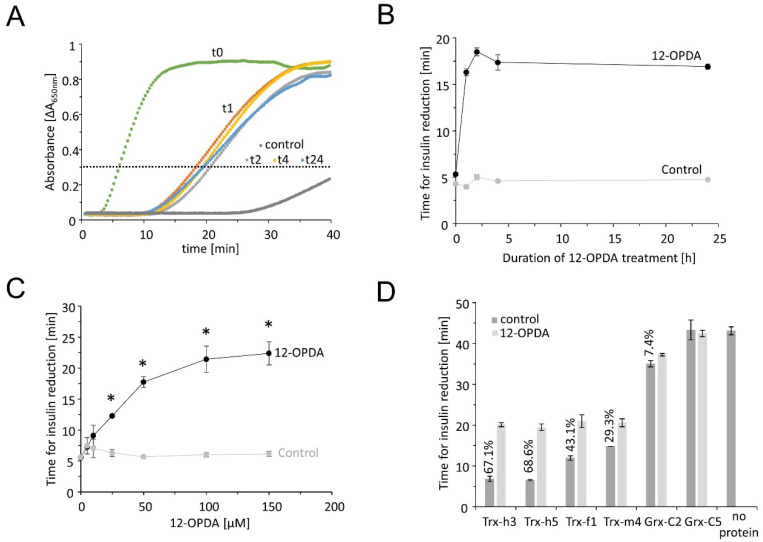
Insulin reduction catalyzed by the redox transmitters TRX-f1, -m4, -h3, -h5 or GRX-C2 with or without 150 µM OPDA pretreatment. Recombinant TRX-f1, TRX-m4, TRX-h3, TRX-h5 and GRX-C2 were reduced and desalted, and either treated with OPDA or the equivalent amount of ethanol as solvent control. In total, 160 µM insulin was placed in the well of a 96-well plate and 5 µM test protein added at t = 0 min. Absorbance increase indicates reduction and precipitation of insulin. (**A**) Exemplary spectroscopic recordings with TRX-h3. The horizontal line indicates the threshold for detailed analysis. Protein was incubated with 12-OPDA for 0 (referred to as t0), 1 (t1), 2 (t2), 4 (t4) or 24 (t24) hours prior to the insulin reduction assay. Additionally, the DTT control is shown in grey. In this case, the absorbance recording remained below the threshold throughout the recorded time span. (**B**) Time-dependent effect of OPDA on reduction activity of TRX-h3. Data are means ± SD of *n* = 5. Time [min] refers to the time needed to reach the threshold of ΔA_650 nm_ = 0.3. (**C**) Concentration-dependent effect of OPDA treatment for 1 h on activity of TRX-h3. Data are means ± SD of *n* ≥ 4. Significance of difference was determined using *t*-test (*p* < 0.001) and is marked with *. (**D**) Comparison of activity of the redox transmitters and inhibitory effect of OPDA [%]. Data are means ± SD of *n* = 5.

Bovine insulin in its oxidized form contains two disulfide bridges that readily accept electrons from TRX [21]. Upon reduction, insulin denatures and tends to aggregate, a process that can be monitored in a spectrophotometer, e.g., multiplexed in a microtiter plate reader. Bovine insulin remained in solution in the absence of TRXs or precipitated very slowly after the addition of low DTT concentrations (Figure 2). Addition of TRX-f1, -m4, -h3 and -h5 accelerated the aggregation rate and the absorbance passed the threshold of ΔA_650 nm_ = 0.3 within a short time period. GRX-C2 was less efficient in insulin reduction than TRXs. In the presence of GRX-C5, the time needed to reach the threshold did not differ from the reaction without any protein. The redox transmitters were incubated with 150 µM OPDA prior to the insulin reduction test. After pretreatment with OPDA, the increase in absorbance and the time period needed to pass the threshold occurred highly delayed, indicating that OPDA binding to the redox transmitters inhibited their disulfide reduction activity and ability to reduce insulin. This effect was scrutinized in a time- and concentration-dependent manner using TRX-h3. After 1 h of OPDA treatment, the threshold was reached after 16.3 ± 0.37 min, compared to the control reaction (TRX-h3 incubated with EtOH), which passed the threshold after 3.98 ± 0.16 min. OPDA extended the time period by a factor of 4 (Figure 2B).

This effect was further characterized by incubating TRX-h3 with different concentrations of 12-OPDA for 1 h (Figure 2C). The inhibitory effect of OPDA on reduction activity was most pronounced for cytosolic TRX-h3 and -h5 followed by chloroplastic TRX-f1 and -m4. Only a small inhibitory effect could be detected on GRX-C2 activity (2D).

#### 3.1.3. Quantification of TRX-OPDAylation

**Figure 3:**
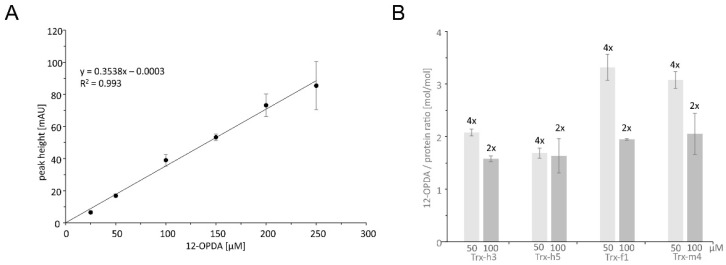
Quantification of OPDA residues bound to TRX-f1, -m4, -h3 and -h5 by HPLC analysis. In total, 200 µM of 12-OPDA was incubated with either 50 or 100 µM of recombinant TRX-h3, TRX-h5, TRX-f1 and TRX-m4 or an equivalent amount of phosphate buffer. Proteins were reduced and desalted prior to the incubation. After 2 h, the amount of free unbound OPDA was determined by reverse phase HPLC. The determined remaining free OPDA was then subtracted from the total amount used for incubation with protein to obtain the amount of OPDA bound to the protein. This value was further divided by the protein concentration to obtain the molar OPDA/protein ratio. (**A**) Different OPDA concentrations were used to generate a standard curve based on peak height [mAU] at the retention time of 14.200 ± 0.014 min. Data are means ± SD of *n* ≥ 3. (**B**) Ratio of OPDA bound to recombinant TRX-f1, TRX-m4, TRX-h3 and TRX-h5 with molar excess of OPDA indicated above each bar. The change in free OPDA concentration was calculated using the standard curve shown in (**A**). Data are means ± SD of *n* ≥ 3.

To quantify the molar stoichiometry of OPDA binding to TRXs, an HPLC-based quantification of OPDA was established. The amount of residual OPDA concentration after reaction with 50 or 100 µM TRX was determined and quantified by comparison with a standard curve (Figure 3A). The molar ratio differed whether OPDA was added in 2-fold or 4-fold excess (Figure 3B) but indicates that two thiols reacted with OPDA in the case of TRX-h3 and TRX-h5 and three thiols in the case of TRX-f1 and TRX-m4. This result supports the conclusion that OPDA efficiently OPDAylates the thiols in TRX proteins.

**Figure 4:**
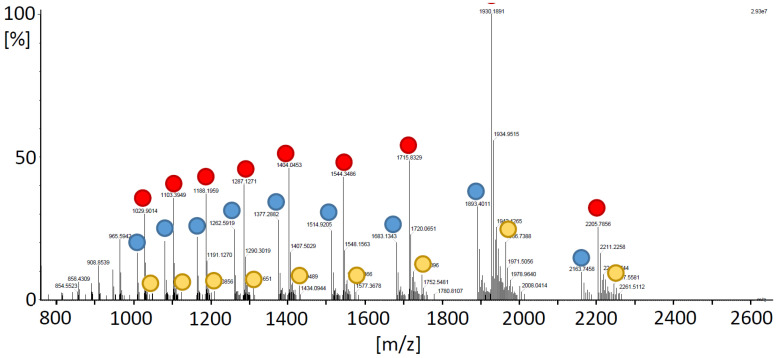
MS analysis of OPDAylated TRX-h3. TRX-h3 (20 µM, in 50 mM NH4Ac) was incubated with 150 µM OPDA overnight and applied to the mass spectrometer. The mass spectrum shows several charge states (z = 7 to 15) of the free TRX-h3 (mass of 15139.12 ± 0.07 Da; blue dots), single OPDA-bound form with a mass of 15,433.43 ± 0.02 Da (red dots) and double OPDAylated form of 15,725.80 ± 0.22 Da (yellow dots).

To validate OPDA attachment by MS, TRX-h3 was OPDAlyated as before, however, in 50 mM ammonium acetate, since the high KP_i_ concentrations used in the other tests might interfere with the MS analysis. Figure 4 depicts the spectra of OPDAylated TRX-h3 and the color-encoded dots mark different charge states (z = 7 to 15) of unlabeled his-tagged TRX-h3 with a deconvoluted mass of 15,139.12 ± 0.07 Da, single OPDAylated form with a mass of 15,433.43 ± 0.02 Da and double OPDAylated TRX-h3 with a mass of 15,725.80 ± 0.22 Da. The unlabeled form lacked 2 atomic mass units indicating the presence of a disulfide bond. The MS analysis was in line with the hypothesized OPDAylation of TRX-h3 and the data shown above.

### 3.2. Inhibition by OPDA of Glutathione Peroxidase-like 8 (GPXL8)-Dependent H_2_O_2_ Reduction

The next step aimed at determining the effect of OPDA on reaction cascades detoxifying reactive oxygen species such as H_2_O_2_ and using redox transmitters such as TRXs as reductants. To this end, we expressed and purified glutathione peroxidase-like 8 (GPXL8) and NADPH-dependent thioredoxin reductase A (NTRA) and TRXs in *E. coli* as above. We reconstituted the pathway by adding NADPH, NTRA, TRX-h3/5 and GPXL8 to the cuvette at protein concentrations aligned with those reported in proteomics work and started the reaction by the addition of H_2_O_2_. Thus, the reconstituted assay used the following cascade of redox reactions:

NADPH + H^+^ → NTRA → TRX-h3/5 → GPXL8 → H_2_O_2_

#### 3.2.1. Optimization of the Enzymatic Test for GPXL8 Activity

**Figure 5:**
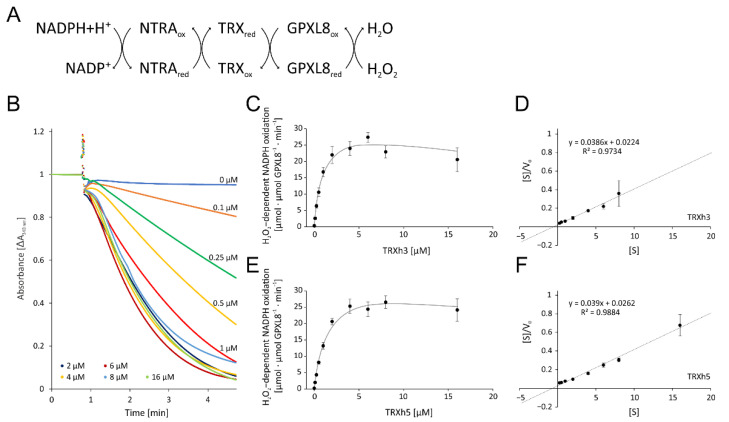
Optimization of the glutathione peroxidase-dependent H_2_O_2_ detoxification assay. (**A**) Scheme of the reaction sequence. (**B**) Representative spectrophotometric recordings adjusted to identical time point of H_2_O_2_ addition and similar baselines of TRXh3 concentration-dependent reduction of H_2_O_2_ by 3 µM GPXL8. The assay contained 1 µM NTRA, 200 µM NADPH, 300 µM H_2_O_2_ and variable TRXh3 concentrations as indicated. The initial linear rate was used for quantification. (**C**) TRXh3 concentration dependency. (**D**) Hanes–Woolf plot of GPXL8 and 0.25–8 µM TRXh3 as electron donor according to (**C**). (**E**) TRXh5 concentration dependency. (**F**) Hanes–Woolf plot of GPXL8 and 0.25–16 µM TRXh5 as electron donor according to (**E**). Data are means ± SD of *n* ≥ 6 (**C**–**F**).

Initially, we determined the optimal reaction conditions and its sensitivity on TRX-amounts (Figure 5). Without the addition of TRX-h3 or -h5, the absorption remained unchanged after the addition of H_2_O_2_ during the 4 min reaction time (Figure 5B). Supplementation with increasing TRX-h3 concentrations enhanced the maximum rate usually observed 10 s after starting the reaction by the addition of H_2_O_2_ (Figure 5C). The K_M_ value was estimated with 0.58 µM TRX-h3 and V_max_ was 25.9 µmol H_2_O_2_ µmol^−1^ protein min^−1^ (Figure 5D). The same analysis was performed with TRX-h5 which gave similar results (Figure 5E) and a slightly higher K_M_ value of about 0.67 µM and a V_max_ of 25.7 µmol H_2_O_2_ µmol^−1^ protein min^−1^ (Figure 5F).

#### 3.2.2. OPDA Effect on TRX-Dependent GPXL8 Activity

**Figure 6:**
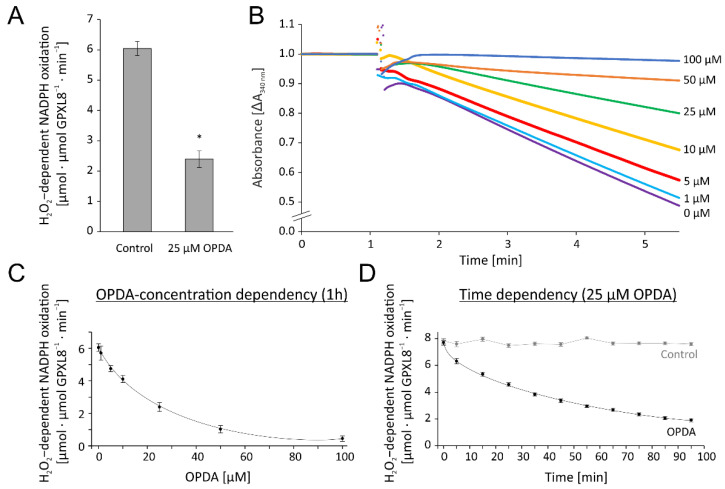
OPDA effect on glutathione peroxidase-like (GPXL)-dependent H_2_O_2_ detoxification using the reconstituted assay. (**A**) Effect of 25 µM OPDA on TRXh3-dependent GPXL8 activity. The reaction mix contained 200 µM NADPH, 1 µM NTRA, 0.25 µM TRX-h3, 3 µM GPXL8 and was incubated for 1h with 25 µM OPDA. After recording a baseline, the reaction was started by the addition of 300 µM H_2_O_2_ at t = 1 min. Significance of difference was determined using *t*-test (*p* < 0.001) and is marked with *. (**B**) Representative absorbance recordings matched to the time point prior to H_2_O_2_ addition and similar baselines of OPDA concentration-dependent activity of GPXL8. The tests were performed in the presence of 0–100 µM OPDA. The control (0 µM OPDA) contained the maximum content of the solvent ethanol according to 100 µM OPDA. Other parameters were as in (**A**). (**C**) OPDA concentration dependency of GPXL8-mediated H_2_O_2_ reduction with 0.25 µM TRX-h3 according to (**B**). (**D**) Time-dependent effect of 25 µM OPDA on GPXL8-mediated H_2_O_2_ reduction with 0.25 µM TRXh3. Other parameters were as in (**A**). Data are means ± SD of *n* ≥ 6 (**A**,**C**,**D**).

The previous results had shown that the reconstituted GPXL8 assay was highly active in detoxifying H_2_O_2._ In an initial test for OPDA effects, we preincubated the reaction mixture with 25 µM OPDA for 1 h or the solvent ethanol as respective control. Their addition to the reconstituted GPXL8 test resulted in an inhibition by 60% (Figure 6A). Based on this preliminary indication for interference of OPDA with the test system and, derived from the above optimization, we opted for 0.25 µM TRX-h3 as the concentration of the redox transmitter in the subsequent tests for the OPDA effect. The recordings presented in Figure 6B,C show the concentration-dependent inhibition of the NADPH-driven H_2_O_2_ reduction after the addition of 10–100 µM OPDA to the cuvette contents 1 h prior to the start of the recording. The kinetics of the inhibition by 25 µM OPDA is shown in Figure 6D. The data show the high efficiency of OPDA to inactivate the test by >50% after 1 h of incubation with 25 µM OPDA (Figure 6D), and half inhibition at approximately 16 µM OPDA after 1 h of incubation (Figure 6C).

#### 3.2.3. Effect of OPDA on Single Test Components and Kinetics of TRX-h3 Inhibition

**Figure 7:**
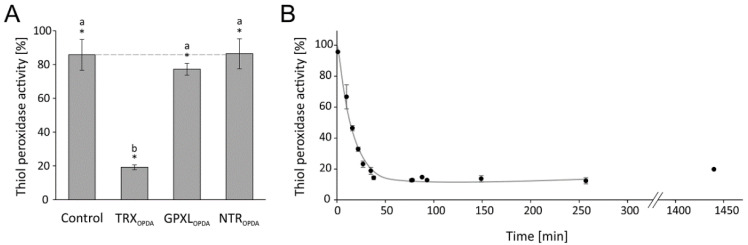
Relative glutathione peroxidase-dependent H_2_O_2_ detoxification after preincubation of single components with OPDA. (**A**) In total, 20 µM reduced and desalted recombinant protein (NTRA, TRX-h3 or GPXL8) was preincubated with 150 µM OPDA for 1h. The pretreated proteins were added to the otherwise untreated assay components as described before: 200 µM NADPH, 1 µM NTRA, 0.25 µM TRX-h3, 3 µM GPXL8 and 300 µM H_2_O_2_. To ensure comparative results, the final OPDA concentration in the reaction mix was 22.5 µM OPDA always. The GPXL8 activity was measured and related to measurements in absence of OPDA but pretreated with the solvent ethanol. Data are means ± SD of *n* = 6. Significance of difference between the components was determined using ANOVA, followed by post hoc Tukey test and is marked with letters a and b. * marks significant differences to corresponding measurements without OPDA as determined by *t*-test (*p* < 0.05). (**B**). In total, 20 µM reduced TRX-h3 was incubated with 150 µM OPDA and the time-dependent effect of OPDA on TRX-h3-dependent GPXL8 activity was measured for 24 h. The assay contained 200 µM NADPH, 1 µM NTRA, 0.25 µM preincubated TRX-h3, 3 µM GPXL8 and 300 µM H_2_O_2_. Data are means ± SD of *n* = 3.

The contribution of single components to the overall inhibition was explored by adding OPDA to NTRA, TRX-h3 and GPXL8 separately before reconstituting the assay. The highest inhibitory effect was seen after treatment of TRX-h3 (Figure 7A). The maximal inhibitory effect on TRX-h3 occured within 40 min and recovers slightly after 24 h (Figure 7B).

### 3.3. OPDA-Dependent Inhibition of Peroxiredoxin IIB-Dependent H_2_O_2_ Reduction

A second thiol peroxidase system is represented by cytosolic peroxiredoxins IIB, C and D [25]. We selected the dominant cytosolic isoform PRXIIB. PRXIIB detoxifies H_2_O_2_ and turns glutathionylated. Highest regeneration rate of active and reduced PRXIIB is achieved by glutaredoxins such as GRX-C2. GRX-C2 reduces the glutathionylated PRXIIB and, with a second glutathione, releases a diglutathione disulfide (GSSG). Glutathione reductase (GR) in turn reduces the GSSG with NADPH+H^+^ as electron donor. Thus, we reconstituted this system with all components from *A. thaliana* as follows:

NADPH → GR → GSH → GRX-C2 → PRXIIB → H_2_O_2_

#### 3.3.1. Optimization of Enzymatic Test for PRXIIB-Dependent H_2_O_2_ Reduction

**Figure 8:**
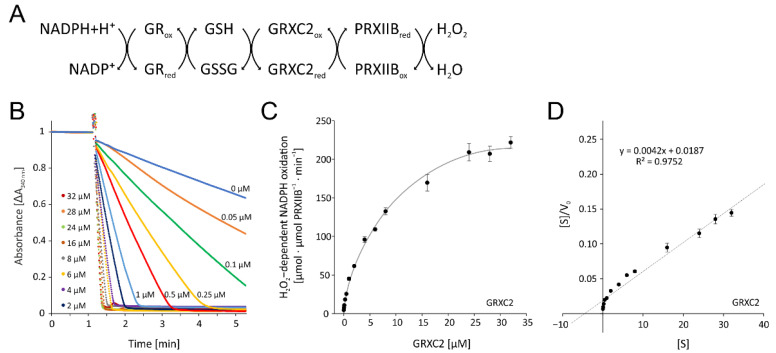
Optimization of the peroxiredoxin IIB-dependent peroxide detoxification assay using the reconstituted system. (**A**) Scheme of the reaction sequence. (**B**) Representative spectroscopic recordings matched to the time point of H_2_O_2_ addition and similar baselines of GRXC2 concentration-dependent reduction of H_2_O_2_ by 3 µM PRXIIB. The assay contained 200 µM NADPH, 1 µM GR, 2 mM glutathione, 3 µM PRXIIB, 300 µM H_2_O_2_ and variable GRXC2 concentrations as indicated. The initial linear rate was used for quantification. (**C**) GRXC2 concentration dependency of H_2_O_2_ reduction by PRXIIB. (**D**) Hanes–Woolf plot of PRXIIB and 0.05–32 µM GRXC2 as electron donor according to (**C**). Data are means ± SD of *n* = 6 (**C**,**D**).

All components were cloned from *A. thaliana*, heterologously expressed in E. coli and purified to high purity. The test was established and optimized for the added GRX-C2 amount (Figure 8B,C). The absorbance recordings show the extremely high rate of NADPH oxidation in the presence of 32 µM GRX-C2 (Figure 8B). Even the lowest tested GRX-C2 concentration of 0.05 µM increased the background rate by 60%. The GRX-C2 concentration dependency was derived from the initial linear rate of NADPH oxidation and revealed a K_M_ value of 4.50 µM and a Vmax of 240.3 µmol H_2_O_2_ µmol^−1^ protein min^−1^ (Figure 8D). The rate of NADPH oxidation without GRXC2 may represent H_2_O_2_-dependent oxidation of glutathione.

#### 3.3.2. OPDA Effect on PRXIIB-Dependent H_2_O_2_ Reduction

**Figure 9:**
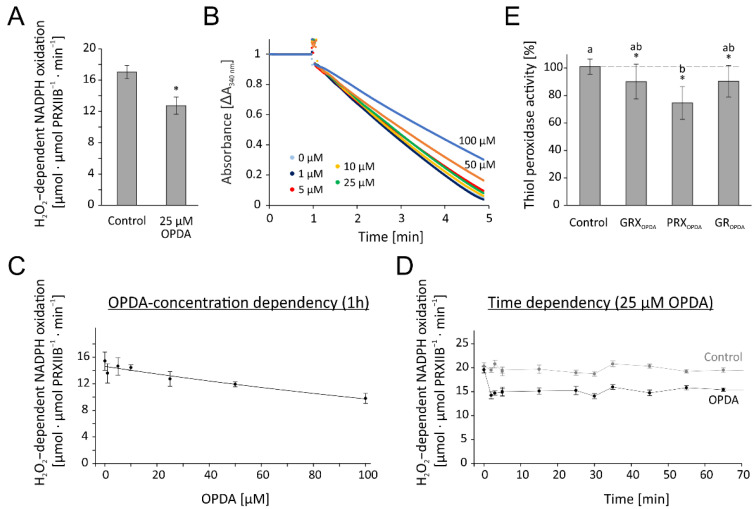
OPDA effect on peroxiredoxin IIB-dependent peroxide detoxification using the reconstituted system. (**A**) Effect of 25 µM OPDA on GRXC2-dependent PRXIIB activity. The reaction mix was incubated for 1 h. The assay was performed with 200 µM NADPH, 2 mM glutathione, 1 µM GR, 0.25 µM GRXC2, 3 µM PRXIIB and 300 µM H_2_O_2_. Data are means ± SD of *n* = 6. Significance of difference was determined using t-test (*p* = 0.0013) and is marked with *. (**B**) Representative absorbance recordings adjusted to the identical time point of H_2_O_2_ addition and similar baselines. The reaction started by the addition of 300 µM H_2_O_2_ at t = 1 min. The tests were performed in the presence of 0–100 µM OPDA. (**C**) OPDA concentration dependency of PRXIIB-mediated H_2_O_2_ reduction with 0.25 µM GRXC2 according to (**B**). Other parameters were as in (**A**). Data are means ± SD of *n* = 6. (**D**) Time-dependent effect of 25 µM OPDA on PRXIIB-mediated H_2_O_2_ reduction with 0.25 µM GRXC2. Other parameters were as in (**A**). Data are means ± SD of *n* ≥ 6. (**E**) Relative glutathione peroxidase-dependent H_2_O_2_ detoxification after preincubation of single components with OPDA. In total, 20 µM reduced and desalted recombinant protein (GR, GRXC2, PRXIIB) was preincubated with 150 µM OPDA for 1 h. The pretreated proteins were added to the otherwise untreated assay components as described in (**A**). To ensure comparative results, the final OPDA concentration within the reaction mix was 22.5 µM OPDA in all assays. The PRXIIB activity was measured and related to results in absence of OPDA but pretreated with the solvent ethanol. Data are means ± SD of *n* ≥ 12. Significance of difference between the components was determined using ANOVA, followed by post hoc Tukey test and is marked with letters a and b. * marks significant differences to corresponding measurements without OPDA as determined by *t*-test (*p* < 0.05).

Initially, the inhibitory effect of 25 µM OPDA on PRXIIB was measured. This concentration was effective in inhibiting GPXL8 activity; however, PRXIIB activity was only slightly affected (Figure 9A). The effect of OPDA on the PRXIIB-dependent H_2_O_2_ detoxification cascade was then further explored by adding various amounts of OPDA in the range of 1–100 µM to the assay. High concentrations of 25–100 µM were required to achieve significant inhibition (Figure 9C). The time-dependent measurement revealed a rapid effect of OPDA on PRXIIB activity within less than five minutes (Figure 9D). To identify the target of OPDA within the PRXIIB peroxidase assay, the single components were preincubated with 150 µM OPDA for 1 h. OPDA seems to act directly on PRXIIB at a low level and only showed an insignificant effect on OPDA-treated GRX and GR (Figure 9E).

### 3.4. Effect of OPDA on Electron Donors of PRXIIB and GPXL8

**Figure 10:**
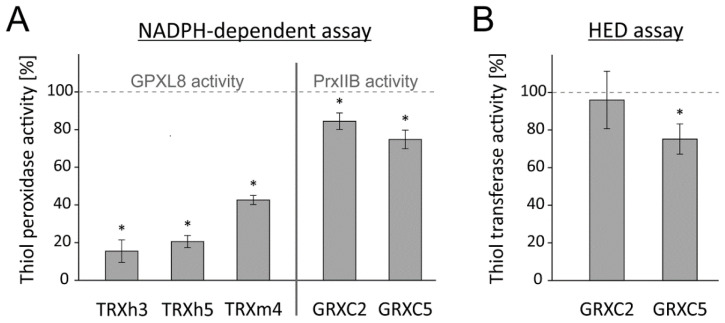
OPDA effect on thiol peroxidase-dependent H_2_O_2_ detoxification using the reconstituted system and on the thiol reduction activity of GRXs. (**A**) In total, 20 µM reduced TRX-h3, TRX-h5, TRX-m4, GRXC2 and GRXC5 were incubated with 150 µM OPDA for 1 h. The GPXL8-dependent activity was measured using TRX-h3 (0.25 µM), TRX-h5 (0.25 µM) or TRX-m4 (5 µM) and the PRXIIB-dependent activity using GRXC2 (0.25 µM) and GRXC5 (0.25 µM), after 1 h preincubation as described before. Data are means ± SD of *n* ≥ 6. Significance of difference was determined using *t*-test (*p* < 0.05) and is marked with *. (**B**) HED assay to determine GRX-dependent reduction activity. In total, 20 µM reduced GRXC2 and GRXC5 were incubated with 150 µM OPDA for 1 h. The activity in the HED assay was measured after 1 h preincubation. The assay contained 200 µM NADPH, 2 mM GSH, 0.7 mM HED, 1 µM GR and 0.1 µM GRXC2. Data are means ± SD of *n* ≥ 12. * marks significant differences to corresponding measurements without OPDA as determined by *t*-test (*p* < 0.001).

Besides the direct impact of OPDA on PRXIIB, the sensitivity of the electron donors TRX-h3, TRX-h5, TRX-m4 and GRXC2, GRXC5 of GPXL8 and PRXIIB, respectively, was investigated by preincubation with OPDA (Figure 10). It turned out that TRXs were more sensitive towards OPDA than GRXs (Figure 10A). All three analyzed TRXs were sensitive to OPDAylation, but the cytosolic TRXs showed an inhibition of roughly 80%, while the chloroplastic TRX-m4 resulted in an inhibition of only 60%. The low sensitivity of GRXC2 and GRXC5 was confirmed by the GRX-specific HED assay, which is independent of the electron acceptor PRXIIB. Here, GRXC2 showed even no significant inhibition by OPDA (Figure 10B). The quantitative differences should be considered with care, because the tests could not be conducted in identical design due to different efficiency of the various redox transmitters in the assays.

### 3.5. OPDA Effect on Glyceraldehyde-3-Phosphate Dehydrogenase GAPC2

**Figure 11:**
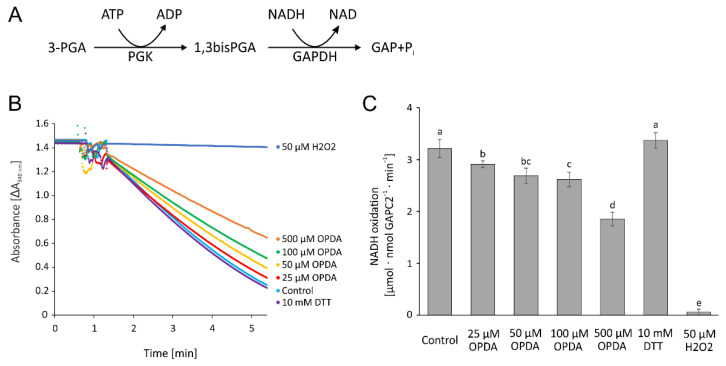
OPDA effect on cytosolic glyceraldehyde-3-phosphate dehydrogenase C2 activity. (**A**) Reaction scheme. (**B**) Representative absorbance recordings normalized to the identical time point of GAPC2 addition and similar baselines after the addition of 15 nM GAPC2. In total, 1 µM reduced and desalted GAPC2 was pre-incubated with 25–500 µM OPDA, 50 µM H_2_O_2_, and 10 mM DTT, respectively, and in the presence of 140 µM NAD for 1 h and then transferred to the GAPC2-activity test. The control containing the equivalent amount of the solvent ethanol was also preincubated for 1 h. The test contained 4 mM ATP, 8 mM 3-phosphoglycerate, 90 nM *A. thaliana* phosphoglycerate kinase, 260 µM NADH+H^+^ and 15 nM GAPC2. (**C**) OPDA concentration-dependent GAPC2 activity as described in (**B**). Data are means ± SD of *n ≥* 6. Significance of difference was determined using ANOVA, followed by post hoc Tukey test and is marked with letters a–e.

Glyceraldehyde-3-phosphate dehydrogenase is a central enzyme in glycolysis, redox homeostasis and carbon metabolism and known for its rather complex regulation and its moonlighting function in gene regulation [26]. To test the effect of OPDA on GAPDH, the coupled activity of phosphoglycerate kinase and GAPDH was measured as consumption of NADH (Figure 11A). Although 25 µM OPDA resulted in significant inhibition, OPDA had a minor impact on the GAPDH activity. The high concentration of 500 µM OPDA reduced the activity by more than 40% (Figure 11B,C).

### 3.6. OPDA Effect on Leaf Tissue

**Figure 12:**
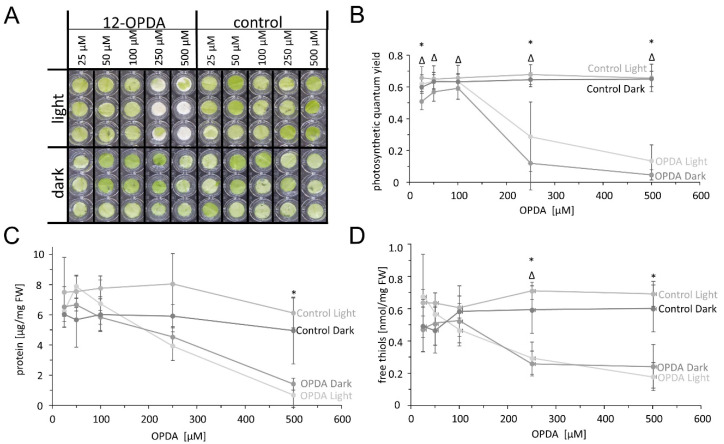
Concentration-dependent effect of OPDA on leaf disc metabolism. (**A**) Exemplary phenotype documentation after 24 h continuous light or darkness. (**B**) Changes in photosynthetic quantum yield of PSII in response to treatment with OPDA. Photosynthetic quantum yield was determined by chlorophyll a fluorescence analysis using the Mini-PAM. Data are means ± SD (*n* = 24). (**C**) Protein and (**D**) free thiol contents were measured after the treatment. Data represent means ± SD of *n* = 3. * and Δ mark significant differences to control in light and dark, respectively, as determined by *t*-test (*p* < 0.05).

To get further insight into the physiological effects of OPDA in vivo, we performed Arabidopsis leaf disc assays. Leaf discs were exposed to OPDA at different concentrations (25, 50, 100, 250 or 500 µM) for 24 h in continuous light or darkness. When subjected to these conditions, the combination of light and high OPDA concentrations (250 and 500 µM) induced a loss of chlorophyll, but no visible damage was observed in the control samples or in the dark (Figure 12A). The leaf status was scrutinized by three parameters. The determination of quantum yield of photosystem II (PSII) revealed a severe concentration-dependent effect of OPDA on the photosynthetic electron transport chain both in light and dark (Figure 12B). Similar patterns were obtained for the contents of protein and free thiols (Figure 12C,D), with the difference that treatment with 250 µM OPDA strongly inhibited the quantum yield of photosystem II (Figure 12B) and lowered the contents of non-protein thiols (Figure 12D), but had much less effect on protein contents.

## 4. Discussion

**Figure 13:**
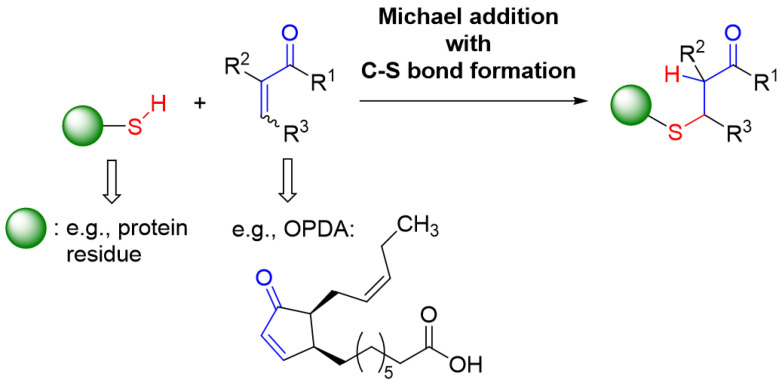
General reaction scheme for the addition of a thiol moiety (e.g., being present in a protein) to an α,β-unsaturated carbonyl compound as a Michael acceptor such as, e.g., OPDA.

Compounds with an α,β-unsaturated carbonyl moiety have electrophilic properties and react with thiols through the addition of the thiols to the C=C double bond of this Michael acceptor and resulting C-S bond formation at the carbon being in β-position of the Michael acceptor (Figure 13).

Their relative reactivity differs depending on additional chemical features. Acrolein and methylvinyl ketone are the smallest compounds with this property that efficiently act as alkylating agents by functioning as effective Michael acceptors. Reaction constants with glutathione decrease from 186 M^−1^s^−1^ for acrolein to 1.59 for crotonaldehyde and 0.52 for cyclopentenone [27]. Cyclopentenone subunits in natural products such as, e.g., OPDA and certain prostaglandins, also contain the reactive α,β-unsaturated carbonyl moiety and, thus, react with thiols, albeit at lower rate [7]. This work scrutinized the efficiency of OPDA to react with protein thiols of the cellular redox regulatory network. Various in vitro assays were conducted. The presented results document and quantify the high reactivity of OPDA with certain thiols, particularly those present in TRXs.

### 4.1. Stoichiometry and Speed of OPDAylation

Validation of OPDA binding to thiols of redox transmitters was first explored by two tests, namely the ability of the redox transmitters to reduce the disulfide bridge in insulin and the covalent binding of mPEGmal (Figure 1 and Figure 2). The obtained banding pattern after mPEG-mal binding to thiols of reduced TRXs was consistent and gave four bands of about 15, 25, 40 and 60 kDa, corresponding to a lack of modification of the cysteinyl residues, a single, double or triple OPDAylation, respectively. Binding of cyclopentenone-containing prostaglandins to human TRX-1 has been observed before [10] and likewise, the first evidence of OPDAylation of TRX-f1 was presented by Maynard et al. [7]. This work expands this previous knowledge to chloroplast TRX-m4 and cytosolic TRX-h3 and -h5.

The same pattern with less intense bands was seen for GRX-C2. To our knowledge, this kind of modification has not been described for GRXs before. Each of these proteins possess three cysteinyl residues. The stepwise increase by about 10 kDa after binding the first mPEGmal molecule, 15 kDa after binding the second and 20 kDa after binding the third was surprising, because the expected shift should comprise 5 kDa due to the size of 5000 Da polyethyleneglycol added to the reactive maleimide; however, unexpected shifts after modification with mPEGmal are not uncommon [28]. Reaction of the maleimide reagent with amine groups, e.g., of lysyl residues on the protein surface by Michael addition is theoretically possible but has not been reported and unlikely is the reason for the shifts, because many lysine residues are solvent accessible and would cause complex shifting patterns.

The reduction assay of insulin disulfides is an established test for redox transmitter activity [21]; however, beyond the evidence for disulfide reduction ability, the test fails to provide physiologically relevant information. The data show that TRXs are efficient and GRXs only have a low disulfide reduction activity since their main function is catalysis of deglutathionylation [29]. GRX-C2 displayed a low disulfide reduction activity which was also sensitive to OPDA treatment, while GRX-C5 was inactive.

OPDA quantification after reaction with TRX gave a third line of evidence for a stoichiometry of two OPDAylated thiols per polypeptide in the case of TRX-h3 and -h5 and three in the case of TRX-f1 and -m4 under close to physiological OPDA concentrations (Figure 3). It should be noted that these assays were conducted with only a 2-fold or 4-fold molar ratio. In the light of three thiols/TRX, the efficiency of OPDA binding to TRX-f1 and -m4 is remarkable, whereas the third thiol of TRX-h3 and -h5 appeared less prone to OPDAylation. Mass spectrometric analysis proved the attachment of up to two OPDA molecules to each TRX-h3 entity (Figure 4).

### 4.2. Specificity of OPDAylation

The OPDA binding stoichiometry detected in the experiments with 2-fold and 4-fold molar ratio of OPDA to proteins already indicated differences in reactivity of thiols within and between proteins. The reconstitution assays of the GPXL8- and PRX-IIB-dependent H_2_O_2_ reduction assay and the activity test of GAPC2 further evidenced the selectivity and specificity of OPDAylation. Plant glutathione peroxidases function as TRX-dependent thiol peroxidases [30,31]. The K_M_ values for TRX-h3 and TRX-h5 of 0.58 and 0.67 µM, respectively, demonstrate the high efficiency of the TRX-GPLXL8 system in detoxifying peroxides. The K_M_ values are in the range of the expected TRX concentrations in the cytosol.

The addition of OPDA to the complete GPXL8 system strongly inhibited the H_2_O_2_ reduction (Figure 6) with measurable differences to control at very low physiological OPDA concentration, namely 1 µM. This experimental setup did not allow for pinpointing to the specific effect of OPDA on each component. The preincubation of individual components revealed that the cytosolic TRX-h3 is particularly sensitive to inactivation by OPDAylation and controls the detoxification rate under these conditions (Figure 7). This is in line with the high sensitivity of human TRX-1 toward inhibition by reacting with the cyclopentenone moiety-containing 15d-PGJ2 [10].

In the PRX-dependent assay, PRX-IIB appeared significantly more prone to OPDAylation and OPDA-dependent inhibition than GRX-C2 and glutathione reductase (Figure 9). However, the extent of inhibition, even at 7.5-fold excess of OPDA, was low in comparison to the effect on TRX. It would be interesting to explore whether other reactive carbonyl species may react with PRXs and may serve as regulators. The 1,3-bisphosphoglycerate oxidation activity of GAPDH displayed OPDA sensitivity as well, but similar to PRX-IIB, the extent of inhibition was rather small and only non-physiologically high OPDA concentrations inhibited GAPC2 to a major extent (Figure 11). This low sensitivity of GAPC2 to OPDA-dependent inhibition surprises in the light of the significant inhibition by H_2_O_2_. However, GAPDH plays additional roles in cell signaling and displays moonlighting functions in gene expression regulation, control of post-transcriptional processes and signaling [32]. Thus, OPDAylation might affect other protein functions, e.g., in protein–protein interactions.

Despite the differences in OPDA sensitivity of various recombinant proteins, these in vitro experiments indicate the high potential of OPDAylation as a significant post-translational modification. Apparently, TRXs and possibly PRXs might be prime targets for this kind of regulation. On the other hand, the here described differential sensitivity of protein thiols to OPDAylation has a high potential for specificity in OPDA-dependent regulation under stress.

### 4.3. OPDA Concentrations and Compartmentation

OPDA is synthesized in the chloroplast and exported to the cytosol and then peroxisome for further processing to jasmonic acid [1,2]. Peroxisomal OPDA reductase 3 (OPR3) is likely key to eliminating the reactive α, β-unsaturated carbonyl group in the cyclopentenone ring [33]. Dueckershoff et al. [8] considered the half life time of OPDA in vivo in the presence of typical glutathione concentrations of 1 mM and suggested a value of about 28 min.

Upon wounding, herbivore attack, high light and other stress impact, OPDA contents increase to several µmol/kg fresh weight. The only important parameter for the here described OPDAylation reaction is the effective concentration of OPDA in the stroma and cytosol. Considering the subcellular volumes and the share of the chloroplast containing mesophyll cells to total fresh weight of leaves, OPDA under stress may be estimated to reach cytosolic and stromal concentrations above 10 µM in mesophyll cells. The assumed half life time of OPDA in the presence of physiological GSH concentrations does not take into account that certain protein thiols may have a particularly high reactivity toward OPDA, as shown in this work for certain TRX and possibly PRX isoforms. Therefore, the release of OPDA may affect redox homeostasis and accumulation of reactive oxygen species in the cell, which may synergistically activate defense programs [12,34].

The strong effect of external feeding of OPDA on the quantum yield of photosystem II, and chlorophyll, protein and free thiol contents of leaf discs is an indication for the strong interference of OPDA with leaf metabolism. Based on the finding of this research, the likely cause for the inhibition of quantum yield even in the dark is the high reactivity of OPDA with thiols and damage to decisive functions in the photosynthetic electron transport chain. The loss of chlorophyll in the illuminated leaf sample indicates that the impaired functionality of the photosynthetic electron transport chain sensitized the chlorophyll for light-dependent bleaching. The bleaching was not seen in the darkened sample despite similar inhibition of photosynthesis.

However, at present it remains unknown whether this effect is due to the conversion of OPDA to jasmonic acid or is caused by the reported functions of OPDA as reactive α,β-unsaturated carbonyl and signaling molecule on its own. Jasmonic acid is reported to induce protein and chlorophyll degradation in some plants such as barley [35]. In any case, there appears to be a threshold between ≤25 and ≥50 µM OPDA where the possible tuning activity, e.g., via OPDAylation of sensitive thiols, turns into a general toxicity as witnessed in the leaf disc assays.

## 5. Conclusions

The covalent association of OPDA with protein thiols, termed OPDAylation, appears to be a potent post-translational modification in plant stress response. The work presented here reveals the high reactivity of OPDA with certain protein thiols in vitro at low physiologically relevant OPDA concentrations. The reported half life time of about 28 min in plant cells needs to be re-evaluated in the light of this high reactivity of certain protein thiols. The subcellular pools of OPDA, i.e., free, electrostatically or covalently bound forms, need deeper scrutiny under stress such as wounding or high light treatment. Our focus on H_2_O_2_-detoxifying thiol peroxidases and the observed inhibition indicate that OPDA-induced accumulation of reactive oxygen species might be linked to OPDAylation of TRX isoforms and peroxiredoxins. The increasing sensitivity of proteomics technology and the improved evaluation methodology for identifying novel post-translational modifications such as OPDAylation under stress provide a promising perspective to gain further insight into the impact of OPDAylation in living plant cells [36]. It will be of interest to compare the relative reactivity of OPDA with other plant TRXs and TRX-fold proteins as well and to elaborate on the features that facilitate OPDAylaton of thiol-containing proteins. This study investigated the spontaneous reaction of protein thiols with OPDA. It would be interesting to explore whether OPDAylation may also be catalyzed by enzymes.

## Data Availability

Data is contained within the article and supplementary material.

## Supplementary Materials

The following supporting information can be downloaded at: https://www.mdpi.com/article/10.3390/antiox11050855/s1, Table S1: Oligonucleotide primers used for molecular cloning of the coding regions of the Arabidopsis proteins. Figure S1: Quantification of band intensities of gel presented as Figure 1.
