# Supplementary material for: OPDAylation of Thiols of the Redox Regulatory Network In Vitro
Source: Antioxidants (Basel). 2022 Apr 27;11(5):855. doi: 10.3390/antiox11050855 (PMC9137622; doi:10.3390/antiox11050855)
Supplement: Supplementary file 1 [file antioxidants-11-00855-s001.zip › antioxidants-1660572-supplementary.pdf]

Supplementary Table S1: Primers used for molecular cloning of coding sequences of genes

Gene	AGI	Vector	Restriction enzyme		Primer Sequence 5'->3'
TRXf1[1]	At3g02730	pET15b	NdeI	Forward	AAAACATATGAGCTTAGAAACCGTTAATGTC
			BamHI	Reverse	TTTTGGATCCTCATCCGGAAGCAGCAGACCTC
TRXm4[1]	At3g15360	pET15b	NdeI	Forward	AAAACATATGGAGGCTCAGGACACCACTG
			BamHI	Reverse	TTTTGGATCCTTACTCGACCAAGAATCTTTCTATAG
TRXh3	At5g42980	pET28a	NdeI	Forward	AAAACATATGGCCGCAGAAGGAGAAGTTATC
			BamHI	Reverse	TTTTGGATCCTCAAGCAGCAGCAACAACTGTCTTG
TRXh5	At1g45145	pET28a	NdeI	Forward	AAAACATATGGCCGGTGAAGGAGAA
			XhoI	Reverse	TTTTCTCGAGTCAAGCAGAAGCTACAAGAC
GRXC2	AT5G40370	pET28a	NheI	Forward	AAAAGCTAGCATGGCGATGCAGAAAGCTAAG
			BamHI	Reverse	TTTTGGATCCTTAAGCAGAAGTTGTTGCAGTCTTTC
GRXC5	AT4G28730	pEXP5-NT/TOPO	TOPO Cloning	Forward	ACTTCTTCTTCTTCGGCTGCATC
				Reverse	AACTCTGACCGTTTTTACCGTTG
GPXL8	At1g63460	pET28a	NdeI	Forward	AAAACATATGGCGACGAAGGAACCAG
			XhoI	Reverse	TTTTCTCGAGTCAGGAGATATTCAGAAGATTCTTTATGTC
NTRA	At2g17420	pET28a	NdeI	Forward	AAAACATATGTCCTCCGCCGCCGCCGCCGT
			BamHI	Reverse	TTTTGGATCCTCAATCACTCTTACCCTCCTGAGATCCAATCTCTTG
PRXIIB	At1g65980	pET28a	NdeI	Forward	AAAACATATGGCTCCAATTGCTGTCGGCGATG
			BamHI	Reverse	TTTTGGATCCTTAGAGAGCCTTGAGGATATCATCGGCGCTG
GR	At3g24170	pET28a	NheI	Forward	AAAAGCTAGCATGGCGAGGAAGATGCTTG
			BamHI	Reverse	TTTTGGATCCTCATAGATTTGTCTTAGGTTTGGGTTTGTG
PGK3	AT1G79550	pET28a	NdeI	Forward	AAAACATATGGCGACGAAGAGAAGCGTTG
			XhoI	Reverse	TTTTCTCGAGTCAAGCTTCGTCGAGAGCGAG
GAPC2[2]	At1g13440	pET16b	NdeI	Forward	GCGCGCCATATGGCTGACAAGAAGATTAG
			BamHI	Reverse	GCGCGCGGATAATTAGGCCTTTGACATGTG

[1] Dreyer, A., Schackmann, A., Kriznik, A., Chibani, K., Wesemann, C., Vogelsang, L., Beyer, A. & Dietz, K. J. (2020). Thiol redox regulation of plant β-carbonic anhydrase. Biomolecules, 10(8), 1125. Reference [17] in main manuscript

[2] Holtgrefe, S., Gohlke, J., Starmann, J., Druce, S., Klocke, S., Altmann, B., Wojtera, J., Lindermayr, C. & Scheibe, R. (2008). Regulation of plant cytosolic glyceraldehyde 3-phosphate dehydrogenase isoforms by thiol modifications. Physiologia Plantarum, 133(2), 211-228. Reference [18] in main manuscript

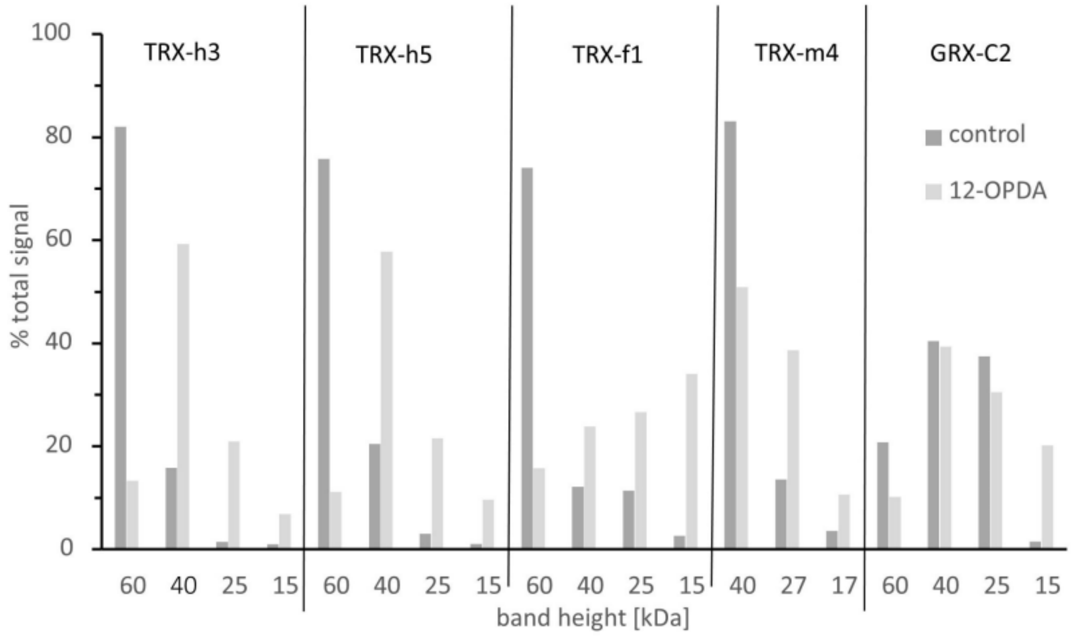

Supplementary Figure S1: Quantification of band intensities of gel presented as Figure 1. Total intensity of all bands in one lane as determined with the image analysis software Image J was set to 100% and percentage of the individual bands calculated as share of total.
